# Boosting Electrooxidation of Ethanol by Nickel Addition to Metallic Glass Ribbon Precursors

**DOI:** 10.3390/ma18030701

**Published:** 2025-02-05

**Authors:** Jingjing Song, Bo Zhang, Yu Chen, Qingzhuo Hu, Fabao Zhang, Langxiang Zhong

**Affiliations:** 1 School of Materials and Chemical Engineering, Bengbu University, Bengbu 233030, China; 2 Songshan Lake Materials Laboratory, Dongguan 523808, China; zhangbo@sslab.org.cn; 3 Anhui Haoou Electronic Technology Co., Ltd., Bengbu 233010, China; 4 School of Materials Science and Engineering, Hefei University of Technology, Hefei 230009, China

**Keywords:** CuNiCe-O composite, metallic glass ribbon, dealloying, ethanol electrooxidation

## Abstract

A CuNiCe-O nanocomposite was fabricated on the Cu_40_Ni_20_Al_10_Ce_26_Pt_3_Ru_1_ metallic glass (MG) ribbon surface by dealloying. The influences of Ni and dealloying time on the morphology and EOR performance were analyzed. The results suggest that the catalytic activity and stability of the dealloyed MG ribbon could be significantly enhanced owing to the alloying of Ni to the Cu_60_Al_10_Ce_26_Pt_3_Ru_1_ MG ribbon precursor. The activated D-Cu_40_Ni_20_Al_10_Ce_26_Pt_3_Ru_1_ ribbon obtained at an etching time of 3 h had a better electrochemical ethanol oxidation reaction (EOR) performance than other dealloyed samples due to the formation of abundant active sites and the presence of defects within the CuNiCe-O composite.

## 1. Introduction

A direct alcohol fuel cell (DAFC) is a low-temperature proton exchange membrane fuel cell that can directly use alcohol aqueous solution as a fuel [1]. Various organic small molecules can be selected as fuels, such as methanol, formic acid, formaldehyde, and ethanol. Among them, ethanol is the simplest chain alcohol, possessing low toxicity. It has a high theoretical energy density (8.1 kWh/kg), low permeability, and a wide range of sources, and can be mass-produced through simple crop fermentation, consistent with the requirements of green chemistry. Therefore, direct ethanol fuel cells (DEFCs) have broad application prospects and great potential to solve energy crises and environmental problems [2].

The research and development of catalysts with high efficiency, low cost, and long service life is very important for the practical application of DEFCs [3]. Electrochemical ethanol oxidation (EOR) plays a key role in electrochemical hydrogen production and DEFCs, both of which are crucial to utilizing renewable energy [2,4]. In general, noble metals such as Pt and Pd have outstanding EOR performances; however, their high cost, scarcity, and easy poisoning by intermediates (such as CO and CH_3_CHO) have always been the main factors restricting the wide application of noble metal-group catalysts [5,6]. Therefore, the design and development of non-noble metal substitutes with high performance and economy have been the focus of researchers [6]. Non-noble-metal-based catalysts, including metal oxides [7], carbides [8], sulfides [9], and nitrides [10], with diverse structures and abundant active sites, improve catalytic activities. Especially, Ni-based electrocatalysts display higher EOR performances [6,7,8]. In particular, Ni oxide [11], Ni hydroxide [12], Ni sulfide [13], Ni nitride [14], and Ni phosphide [15] were developed for the electrooxidation of ethanol. In recent years, the focus of research has been on understanding the reaction chemistry and mechanism, the determination of active sites, and the design of catalysts [16,17,18]. Various strategies, such as promotion with non-noble transition metals [19,20], promotion with noble metals [21,22,23], amorphization [24], and promotion with carbon materials [25], have been utilized to develop and improve the activities of Ni-based catalysts for efficient EOR. These strategies have enhanced the specific surface area, the number of active sites, synergetic effects, conductivity, and mass transfer, remarkably enhancing EOR performance [26].

Amorphous alloys also known as metallic glass (MG) have a uniform microstructure with long-range disorder up to the sub-nanometer scale. A great number of reports have shown that nanoporous materials obtained by dealloying metallic glass precursors have a more uniform structure and composition [27,28]. Furthermore, metal oxides fabricated by dealloying have better morphology and catalytic properties than those dealloyed from the corresponding crystalline alloys [29,30]. Ni can activate water and provide more OH_ads_ (H_2_O → OH_ads_ + H^+^ + e) for catalysis, which helps to completely oxidize intermediates and improve the catalysts’ resistance to poisoning [31]. At the same time, the existence of Cu is beneficial to the formation of β-NiOOH with a higher level of activity [32]. In this work, Ni was introduced into a Cu_60_Al_10_Ce_26_Pt_3_Ru_1_ MG ribbon, and a CuNiCe-O nanocomposite was constructed on the surface of the Cu_40_Ni_20_Al_10_Ce_26_Pt_3_Ru_1_ MG ribbon. The effects of Ni addition to the MG ribbon precursor and dealloying time on the morphology and EOR performance were studied. The results suggest that the dealloyedCu_40_Ni_20_Al_10_Ce_26_Pt_3_Ru_1_ ribbon had a better EOR performance than the dealloyed Cu_60_Al_10_Ce_26_Pt_3_Ru_1_ ribbon due to the synergy of Cu and Ni oxides and the formation of abundant active sites and defects within the dealloyed layer composites of the AD-Cu_40_Ni_20_Al_10_Ce_26_Pt_3_Ru_1_ ribbon.

## 2. Materials and Methods

### 2.1. Chemicals

Potassium hydroxide (KOH) and nitric acid (HNO_3_) were purchased from Sinopharm Chemical Reagent Co., Ltd., Shanghai, China. Deionized water was used for solution preparation. All chemical reagents were analytically pure and were used without further purification.

### 2.2. Preparation of MG Ribbons and Dealloyed Electrocatalysts

The preparation of Cu_60_Al_10_Ce_26_Pt_3_Ru_1_ and Cu_40_Ni_20_Al_10_Ce_26_Pt_3_Ru_1_ MG ribbons is outlined. First, it was necessary to calculate the mass of the required high-purity Ce, Al, and Cu metals (purity over 99.95, wt%) according to the atomic ratio of MG ribbons. Next, we weighed the metal to the nearest ± 0.0005 g. Subsequently, under the protection of argon, the Ti ingot was melted several times to remove residual air, and then the metals were melted 3–5 times into alloy ingots with uniform components. After that, we placed the alloy ingot into a high-purity quartz tube, heating it by electromagnetic induction. The molten alloy was then sprayed onto a copper roller, rotating at a high speed, and thrown out [33]. An MG ribbon was obtained with a width of about 1.5 mm and a thickness of about 20 μm.

**Figure 1:**
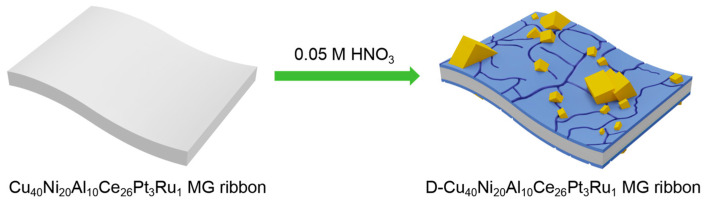
A schematic diagram of the D-Cu_40_Ni_20_Al_10_Ce_26_Pt_3_Ru_1_ MG ribbon produced by dealloying.

The preparation of dealloyed samples proceeded as follows. Dealloying was performed at normal temperatures and pressures by putting the amorphous alloy ribbons in 0.05 M HNO_3_. Then, the dealloyed MG ribbon obtained was washed with water and alcohol more than 3 times and dried naturally (marked as D-MG ribbon), as shown in Figure 1. After that, the activation of the as-dealloyed products was carried out by the cyclic voltammetry (CV) scanning method through 100 scan cycles at 50 mV/s in the potential range from −0.8 to 1.0 V vs. Hg/HgO in a 1 M KOH aqueous solution. Then, the electrode material (AD-MG ribbon) was prepared.

### 2.3. Structure Characterization

XRD (Rigaku XR, Cu-Kα, Tokyo, Japan) was used to identify the phase structure. The voltage and current were 40 kV and 30 mA, respectively. Morphology and microstructure characterization were performed using field emission scanning electron microscopy (FE-SEM, Zeiss Auriga, Gemini 500, Oberkochen, Germany) and transmission electron microscopy (TEM, Japan-JEM-2100F, Tokyo, Japan). The valence states and binding energies of element catalysts were analyzed by an X-ray photon energy spectrometer (XPS, Thermo ESCALAB250Xi, Waltham, MA, USA) equipped with Al-Kα (hν = 1486.6 eV) radiation and calibrated internally by a carbon deposit C 1s binding energy of284.8 eV.

### 2.4. Electrochemical Test

Electrochemical measurements were performed using the Shanghai Chenhua Electrochemical Workstation (CHI 760E, Shang Hai, Shanghai, China). A classical three-electrode system was used, wherein the dealloyed MG ribbons were utilized as working electrodes, saturated Hg/HgO (1 M KOH) was used as the reference electrode, a Pt sheet (0.5 cm × 1.0 cm) was used as the counter electrode, and the electrolyte solution was 1.0 M KOH or 1.0 M KOH + 1.0 M ethanol (1.0 M KOH containing 1.0 M ethanol). The working electrode area was kept at (0.15 × 2) cm^2^, and the rest was sealed with acrylate adhesive. N_2_ was bubbled through the electrolyte for 20 min before electrochemical testing.

Before electrochemical testing, the dealloyed ribbons (D-MG ribbons) were activated in a 1.0 M KOH electrolyte. Then, the activated dealloyed MG ribbons (AD-MG ribbons) were then used directly in the electrocatalytic ethanol oxidation. CVs were conducted at scan rate of 30 mV/s. Chronoamperometric tests (CAs) were performed at 1.57 V (vs. RHE) for 10 h. The potentials were converted into that potential relative to the standard hydrogen electrode by using Formula (1):

(1) $$E\left(RHE\right)=E\left(Hg•HgO\right)+0.921$$

The current density was normalized using Equation (2).

(2) $$j\left(mA\;cm^{-2}\right)=i/S$$

where *i* is the current (mA) and *S* is the effective area of the working electrode immersed in the electrolyte.

## 3. Results

### 3.1. EOR Performance

**Figure 2:**
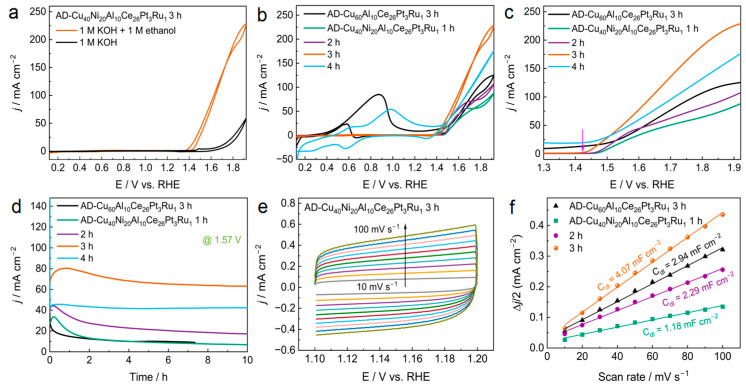
The EOR performance of all samples. (**a**) CV curves of AD-Cu_40_Ni_20_Al_10_Ce_26_Pt_3_Ru_1_-3 h ribbon utilized in 1.0 M KOH and 1.0 M KOH + 1.0 M ethanol aqueous solution. (**b**) CV curves of all samples recorded in 1.0 M KOH + 1.0 M ethanol solution. (**c**) The enlarged view of the forward scan in (**b**) (The purple arrow represents the onset potential of AD-Cu_40_Ni_20_Al_10_Ce_26_Pt_3_Ru_1_-3 h ribbon). (**d**) CA curves. (**e**) CVs of AD-Cu_40_Ni_20_Al_10_Ce_26_Pt_3_Ru_1_-3 h ribbon performed in 1.0 M KOH at different scan rates in a potential window where no Faradaic processes occur (CVs potential range of AD-Cu_40_Ni_20_Al_10_Ce_26_Pt_3_Ru_1_ ribbon was 1.10–1.20 V and that of AD-Cu_60_Al_10_Ce_26_Pt_3_Ru_1_ was 1.2–1.3 V, different color lines represent different scanning rates from 10 to 100 mV/s.). (**f**) Charging current density differences (Δj/2 = (ja − jc)/2) plotted against scan rates: the linear slope equivalent to the double-layer capacitance, C_dl_, was used to represent the ECSA. CV scan rate was 30 mV/s, and the CA test was conducted at 1.57 V.

EOR was conducted in an alkaline solution to assess the electrocatalytic performance of the catalysts. The EOR performance of the as-spun alloy ribbons was also investigated and the results obtained revealed that they exhibited negligible electrocatalytic activity toward EOR (Figure S1). Figure 2 shows the EOR performance of the dealloyed samples. As can be seen from Figure 2a, when ethanol was not added, there was a pair of redox peaks in the CV spectrum between 1.4 and 1.6 V, which was Ni^2+^↔Ni^3+^ redox. When ethanol was added, a strong oxidation peak appeared after 1.3 V, indicating that ethanol was oxidized [34]. Furthermore, as shown in Figure 2b, oxidation peaks appeared in the positive and negative scans in the range of 0.3~1.3 V in the CV spectrum of the AD-Cu_60_Al_10_Ce_26_Pt_3_Ru_1_-3 h MG ribbon. These are typical oxidation peaks of Pt-catalyzed ethanol oxidation. Strong oxidation peaks at 1.3~1.921 V are attributed to the ethanol oxidation by the surface products of the dealloyed MG ribbons. When the CV spectrum of the AD-Cu_40_Ni_20_Al_10_Ce_26_Pt_3_Ru_1_ MG ribbon was observed, only the oxidation peak of Pt to ethanol appeared in the CV spectrum of the AD-Cu_40_Ni_20_Al_10_Ce_26_Pt_3_Ru_1_-4 h ribbon, whereas the other three samples only showed oxidation peaks in the range of 1.3~1.921 V, indicating that Pt active sites were not formed on the surface of the AD-Cu_40_Ni_20_Al_10_Ce_26_Pt_3_Ru1-1 h, -2 h, or -3 h MG ribbons. Figure 2d shows that the AD-Cu_60_Al_10_Ce_26_Pt_3_Ru_1_-3 h ribbon without Ni is the most unstable for catalytic ethanol oxidation amongst those tested. Moreover, the C_dl_ of the AD-Cu_40_Ni_20_Al_10_Ce_26_Pt_3_Ru_1_-3 h ribbon was 4.07 mF cm^−2^, which was higher than that of the AD-Cu_60_Al_10_Ce_26_Pt_3_Ru_1_-3 h ribbon (C_dl_ = 2.94 mF cm^−2^), demonstrating the larger electrochemical active surface area of the CuNiCe-O nanoparticle constructed on the AD-Cu_40_Ni_20_Al_10_Ce_26_Pt_3_Ru_1_-3 h ribbon surface (Figure 2e,f). Therefore, this suggests that the catalytic activity and stability of the activated dealloyed MG ribbons can be enhanced when Ni is introduced into the MG ribbon precursors. The stability of the AD-Cu_40_Ni_20_Al_10_Ce_26_Pt_3_Ru_1_ ribbon was the highest, and the current density decreased by only 5% after the CA test for 10 h. Nevertheless, the current density increased at the beginning of the CA curve, probably because the activated sample was used in 1 M KOH, although CV activation tended to stabilize; however, in the CA test, due to the test being performed at the high potential of 1.57 V (vs. RHE), the surface of the sample continued to react, and the current density reached the maximum valid and then began to decrease. Figure 2c and Table 1 show that the AD-Cu_40_Ni_20_Al_10_Ce_26_Pt_3_Ru_1_-3 h ribbon has the lowest onset potential (1.437 V), indicating that its catalytic efficiency is the highest [35].

### 3.2. Morphological and Structural Characterization

**Figure 3:**
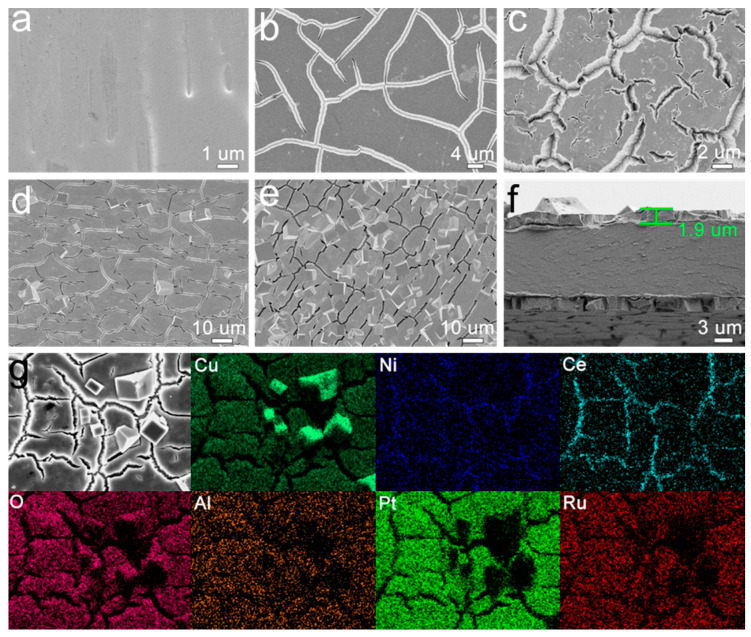
Surface morphology of D-Cu_40_Ni_20_Al_10_Ce_26_Pt_3_Ru_1_ ribbon: (**a**–**e**) surface SEM of D-Cu_40_Ni_20_Al_10_Ce_26_Pt_3_Ru_1_ with etching times of 0, 1, 2, 3, and 4 h; (**f**) SEM cross-sectional image of D-Cu_40_Ni_20_Al_10_Ce_26_Pt_3_Ru_1_-3 h ribbon; (**g**) SEM-mapping image of D-Cu_40_Ni_20_Al_10_Ce_26_Pt_3_Ru_1_-3 h ribbon.

The approximate content of elements contained in the as-spun (as-prepared) Cu_60_Al_10_Ce_26_Pt_3_Ru_1_ and Cu_40_Ni_20_Al_10_Ce_26_Pt_3_Ru_1_MGribbonsis shown in Figure S2, and it is consistent with the theoretical content. As can be seen from Figure 3, the surface of the Cu_40_Ni_20_Al_10_Ce_26_Pt_3_Ru_1_ MG ribbon is relatively smooth (Figure 3a). When the dealloying time was 1 h and 2 h, a flat and cracked oxide layer appeared on the surface of the MG ribbon (Figure 3b,c). When the dealloying time was extended to 3 h, a small number of cubic grains were formed on the surface of the dealloyed layer (Figure 3d). When the dealloying time was 4 h, the cubic grains increased obviously in amount (Figure 3e). As can be seen from Figure 3f, the thickness of the dealloyed layer of the D-Cu_40_Ni_20_Al_10_Ce_26_Pt_3_Ru_1_-3 h MG ribbon was about 1.9 μm. Figure 3g shows that the components of the dealloyed layer and cubic crystal grains were mainly Cu and O. The EDS result (Figure S3) shows that the atomic ratio of Cu and O in the cracked dealloyed layer was less than 1:1, indicating that it was mainly composed of CuO. The atomic ratio of Cu and O in the cubic particles was close to 2:1, demonstrating that the composition of cubic particles was Cu_2_O [36].

**Figure 4:**
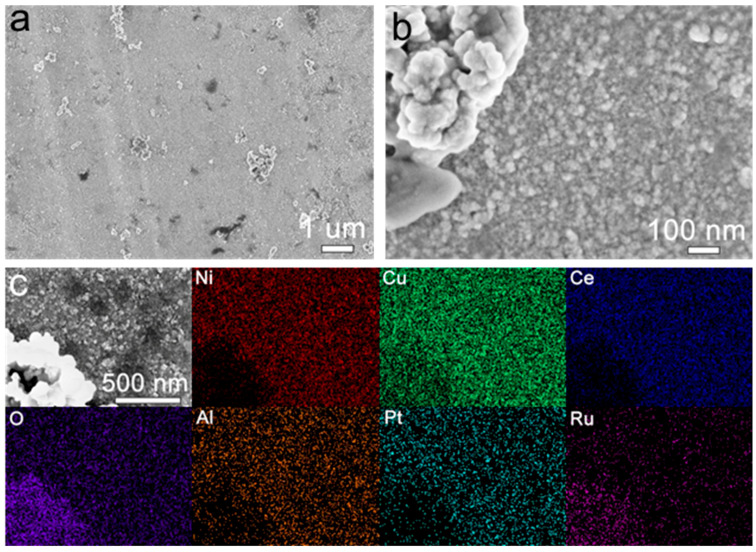
The surface morphology of the AD-Cu_40_Ni_20_Al_10_Ce_26_Pt_3_Ru_1_-3 h ribbon: (**a**) an SEM image; (**b**) an internal SEM image of (**a**); (**c**) SEM-mapping image.

The EOR performance showed that only the activated dealloyed MG ribbon had catalytic activity; compared to the other samples, the AD-Cu_40_Ni_20_Al_10_Ce_26_Pt_3_Ru_1_-3 h ribbon exhibited an outstanding performance. The surface SEM images of the AD-Cu_40_Ni_20_Al_10_Ce_26_Pt_3_Ru_1_-3 h ribbon are shown in Figure 4. Figure 4a,b show that the surface of the activated sample is mainly covered by nanoparticles, except for a small number of irregular bulk structures. As can be seen from Figure 4c, the two structures above are mainly composed of Cu, Ce, Ni, and O, which are evenly distributed, indicating that the CuNiCe-O composite is formed on the surface of the AD-Cu_40_Ni_20_Al_10_Ce_26_Pt_3_Ru_1_-3 h MG ribbon. It can be seen that the Ce (27.4%), Cu (26.0%), Ni (17.5%), and O (17.3%) atomic ratio of nanoparticles is relatively high (Figure S4). The irregular bulk structures have lower Ni (9.2%) and higher Ru (8.4%) and O (40.9%) contents.

The surface SEM image of the AD-Cu_40_Ni_20_Al_10_Ce_26_Pt_3_Ru_1_-3 h ribbon after the CA test for 10 h is shown in Figure S5. It can be seen that a small number of island-like and aggregated nanosheet structures appear on the ribbon surface (Figure S5a). As can be seen from the enlarged view, nanosheets are distributed on the island and substrate surfaces (Figure S5b,c). The EDS spectra show that the islands are mainly composed of Pt-CuO, while the nanosheets are mainly composed of Cu, O, Ce, and Ni. The structural transformation after activation may be related to the increase in current density at the beginning of the CA test, resulting in the nanoparticle structures being transformed into nanosheets and promoting the enrichment of Pt to form the Pt-rich island structure.

**Figure 5:**
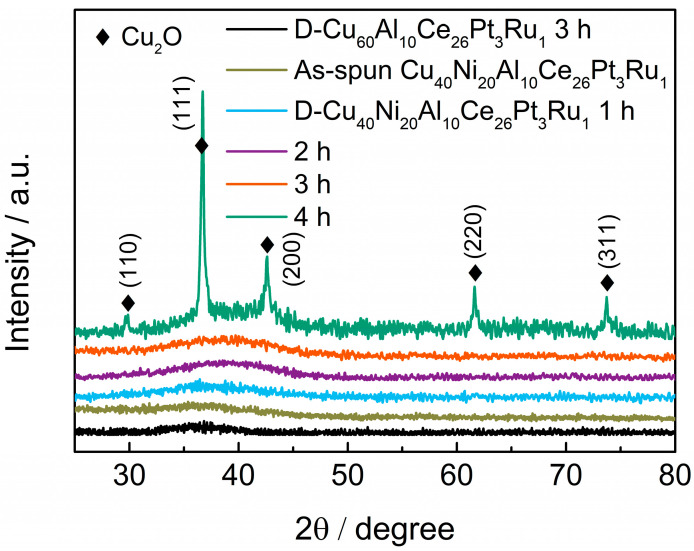
The XRD patterns of all samples.

The XRD pattern of the samples is shown in Figure 5. It can be seen from the figure that the D-Cu_60_Al_10_Ce_26_Pt_3_Ru_1_, Cu_40_Ni_20_Al_10_Ce_26_Pt_3_Ru_1_, D-Cu_40_Ni_20_Al_10_Ce_26_Pt_3_Ru_1_ (dealloying time t = 1–3 h), and AD-Cu_40_Ni_20_Al_10_Ce_26_Pt_3_Ru_1_-3 h ribbons only have one broad peak in the XRD diffraction spectrum, located between 30 and 45°, indicating that their phase structure is amorphous. From the SEM analysis results (Figure 3), it is observed that although a layer of metal oxide was formed on the surface of the dealloyed samples, the thickness of the surface product was no more than 2 μm (Figure 3f), while the thickness of the sample measured by XRD diffraction was generally no less than 5 μm [37]. Therefore, for the dealloyed and activated samples, the XRD tests were performed on the amorphous alloy inside, and so they still showed amorphous structures. Although a small number of cubic particles with large sizes were generated on the surface of the D-Cu_40_Ni_20_Al_10_Ce_26_Pt_3_Ru_1_-3 h ribbon (Figure 3d), the XRD diffraction spectrum did not detect the corresponding crystal peaks due to their small number and as such still presented an amorphous phase. However, when the dealloying time was 4 h, the cubic particles increased obviously and almost covered the oxide layer (Figure 3e). Therefore, both amorphous structure diffraction peaks and strong Cu_2_O crystal peaks appeared in the XRD diffraction spectrum (Figure 5). The diffraction peaks at 29.83°, 36.71°, 42.57°, 61.60°, and 73.70° were indexed to the (110), (111), (200), (220), and (311) planes of Cu_2_O (PDF No. 05-0667).

**Figure 6:**
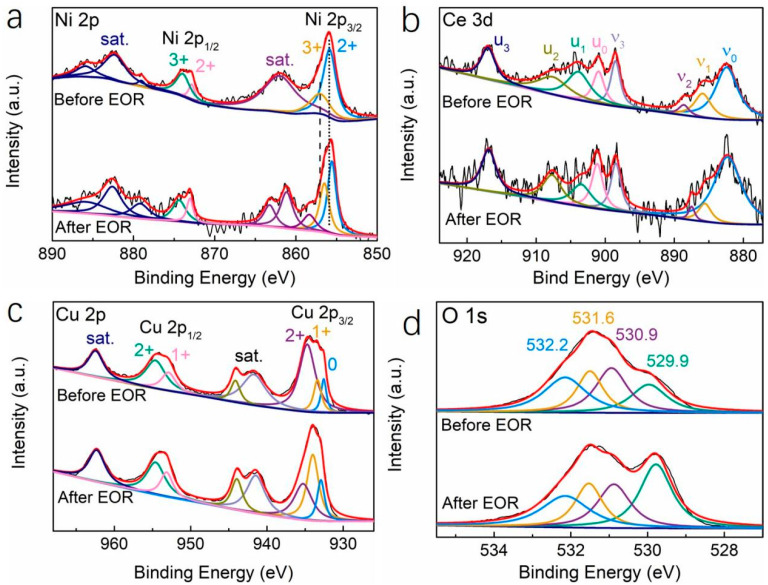
XPS spectra of the AD-Cu_40_Ni_20_Al_10_Ce_26_Pt_3_Ru_1_-3 h ribbon before and after EOR: (**a**) Ni 2p, (**b**) Ce 3d, (**c**) Cu 2p, and (**d**) O 1s.

To identify the valence state of the surface product of the AD-Cu_40_Ni_20_Al_10_Ce_26_Pt_3_Ru_1_-3 h ribbon, an XPS test was performed and the results are shown in Figure 6. Figure 6a shows that two clusters of spin–orbit peaks appear in the high-resolution Ni 2p spectrum, accompanied by a cluster of resonant satellite peaks, corresponding to Ni 2p3/2 and Ni 2p1/2, respectively. Specifically, two peaks at ~855.8 and 872.8 eV correspond to Ni^2+^, and two peaks at ~857.0 and 873.8 eV are attributed to Ni^3+^ [38]. In addition, the spectra of Ni 2p3/2 and Ni 2p1/2 show that the strong satellite peaks with high binding energy located at 860–870 eV and 877–890 eV are split, indicating that they have multi-electron excitation characteristics [39]. In Figure 6b, two sets of spin–orbit multiplet peaks labeled (u_0_–u_3_) and (v_0_–v_3_) correspond to Ce 3d3/2 and Ce 3d5/2 in the Ce 3d spectrum, respectively. Peaks labeled v_0_, v_2_, v_3_, u_0_, u_2_, and u_3_ indicate the presence of Ce^4+^ species (CeO_2_), while peaks labeled v_1_ and u_1_ indicate the presence of Ce^3+^-containing components (Ce_2_O_3_) [40,41,42]. Calculation shows that the peak area of the Ce^3+^ component accounts for about 28%, indicating that there are many unsaturated Ce^3+^components in the surface product. The existence of Ce^3+^ means that the catalyst contains a large number of O vacancies, which can greatly enhance the catalytic activity and stability of the catalyst [43]. In Figure 6c, from Cu 2p spectra, it is obvious that the two main peaks at 934.7 eV and 954.5 eV, together with satellite peaks at 941.7 eV and 944.1 eV, are attributed to Cu^2+^ (CuO).The two peaks at 933.4 eV and 952.8 eV correspond to Cu^+^, and the other peak at 932.6 eV can be attributed to CuO [44,45]. As shown in Figure 6d, two band peaks at ~529.9 eV and 530.9 eV are attributed to lattice oxygen (O_Lattice_) of metal oxides. The peak at 531.6 eV corresponds to an O vacancy, and the peak at 532.2 eV corresponds to adsorbed O, such as the adsorption of H_2_O from the atmosphere [46,47,48]. The XPS results are consistent with SEM.

**Figure 7:**
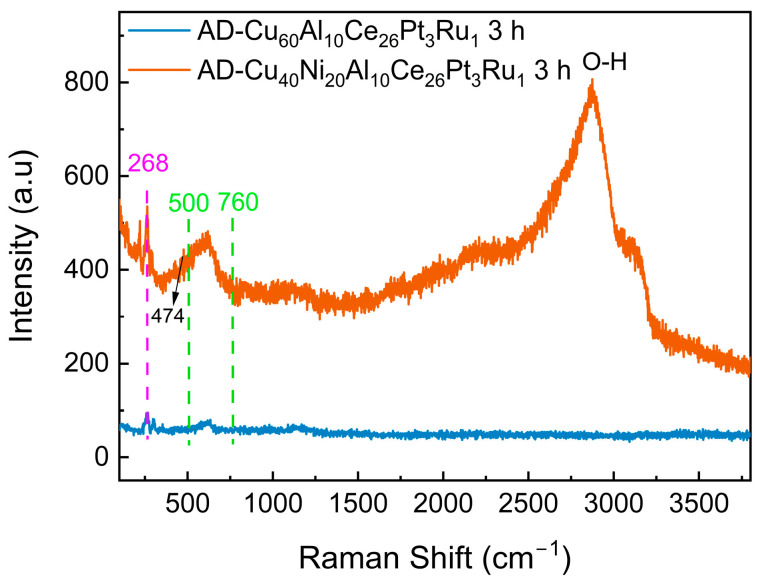
Raman spectra of the AD-Cu_40_Ni_20_Al_10_Ce_26_Pt_3_Ru_1_-3 h and AD-Cu_60_Al_10_Ce_26_Pt_3_Ru_1_-3 h ribbons.

The Raman spectra of the AD-Cu_40_Ni_20_Al_10_Ce_26_Pt_3_Ru_1_-3 h and AD-Cu_60_Al_10_Ce_26_Pt_3_Ru_1_-3 h ribbons are shown in Figure 7. The peaks at 268 and 474 cm^−1^ correspond to the bending vibrations of the Ce-O and Ni-O bonds, respectively [49,50]. The band spanning from 500 to 760 cm^−1^ can be attributed to the nondegenerate LO mode of ceria related to the oxygen vacancies in the ceria lattice [51]. Generally, the wider this band (F2g), the greater the number of oxygen vacancies [52,53]. The band located at 2500–3500 cm^−1^ is attributed to O-H vibrations, demonstrating that the surface product of the AD-Cu_40_Ni_20_Al_10_Ce_26_Pt_3_Ru_1_-3 h ribbon contains abundant hydroxyl groups. Thus, the obtained XPS and Raman data confirm the presence of more defects/oxygen vacancies in the dealloyed layers of the AD-Cu_40_Ni_20_Al_10_Ce_26_Pt_3_Ru_1_-3 h ribbon than in those of the AD-Cu_60_Al_10_Ce_26_Pt_3_Ru_1_-3 h ribbon, which promotes the adsorption of OH groups, oxygen ions, and other oxygen-containing species, boosting EOR performance [39].

### 3.3. Dealloying Reaction and EOR Mechanism

It can be seen from Table 2 that the Ce content in the acid solution after dealloying is the highest, followed by Cu, Ni, and Al. According to the above analysis of the composition and morphology, it can be inferred that the possible reactions in the dealloying process are as follows:

(3) $$2Ce+Cu+6H^{+}+H_{2}O\to 2{Ce}^{3+}+CuO+4H_{2}$$

(4) $$CuO+2H^{+}\to {Cu}^{2+}+H_{2}O$$

(5) $$Ni+2H^{+}\to {Ni}^{2+}+H_{2}$$

(6) $$2Al+6H^{+}\to 2{Al}^{3+}+3H_{2}$$

From the above analysis, it can be observed that the CuO layer is mainly formed on the MG ribbon surface after dealloying, and that Pt atoms migrate to the surface at the same time. From the EDS test results shown in Figure 3g, it can be seen that the Pt content in the dealloyed oxide layer is about 9.7%, which is larger than the amount in the MG ribbon precursor. The CuNiCe-O oxide layer is formed by the recombination of elements during CV activation. Pt atoms may be covered, resulting in lower Pt in the surface products (Figure S4). The mechanism of the ethanol oxidation reaction on the CuNiCe-O composite surface in an alkaline environment can be speculated to be as follows:

(7) $${Cu}^{2+}↔{Cu}^{3+}+e$$

(8) $${Cu}^{3+}+CH_{3}CH_{2}OH_{ads}+Intermediate+{Cu}^{2+}$$

(9) $${Cu}^{3+}+Intermediate\to Product+{Cu}^{2+}$$

It has been reported that doping Cu into Ni-based catalysts can increase the percentage of high-valence Ni (Ni^3+^). In addition, the introduction of Cu increases the specific surface area and produces more defect sites, enhancing EOR performance [54].

(10) $${Ni}^{II}↔{Ni}^{III}+e$$

(11) $${Ni}^{III}+ethanol\to Intermediate+{Ni}^{II}$$

(12) $$Intermediate+{Ni}^{III}\to Product+{Ni}^{II}$$

The electrocatalytic oxidation mechanisms of Ni and Cu are similar for ethanol. However, it can be seen from Figure 2b,c that after Ni is introduced into the precursor, the activity and stability of the activated dealloyed MG ribbon are significantly improved, indicating that Ni plays a crucial role in enhancing catalysis. The Cu and Ni composite can fill the d band vacancy of Ni and inhibit the formation of γ-NiOOH. The formation of a high-valence-state γ-NiOOH competes with β-NiOOH, and the latter shows higher catalytic activity under alkaline conditions [32,55,56]. The surface catalytic activity and excellent electrical conductivity of nickel are very important for improving EOR performance. The state of Ni, especially the +3 state NiOOH, has high electrical activity, which improves reaction efficiency [57]. Ni and Ce ions which are not saturated can also provide a large number of oxygen vacancies, which make ethanol molecules and hydroxyl components easy to adsorb onto the catalyst surface, thereby reducing the oxidation potential of ethanol and oxidizing the intermediates in time so that the active sites can be quickly released. Hence, the activity and stability are enhanced. As can be seen from Figure 6a,c, after the catalyst is used, the energy bands of Ni^2+^and Ni^3+^ move to a lower binding energy, and the percentage of Cu^2+^ decreases. From Table S1, it can be observed that the percentage content of adsorbed oxygen (532.2 eV) is obviously reduced after the reaction. These results indicate that Ni, Cu, and -OH_ads_ play a key role in the catalytic reaction [58,59].

## 4. Conclusions

In this work, Ni was introduced into Cu_60_Al_10_Ce_26_Pt_3_Ru_1_ MG, and the relationship between the morphology of D/AD-Cu_40_Ni_20_Al_10_Ce_26_Pt_3_Ru_1_ MG ribbons and EOR performance was investigated. The results show that introducing Ni into the MG ribbon precursor may change the migration paths of elements during dealloying, resulting in the formation of CuCeNi-O composite nanoparticles on the surface of the activated dealloyed MG ribbons. Compared with the AD-Cu_60_Al_10_Ce_26_Pt_3_Ru_1_ ribbon, the AD-Cu_40_Ni_20_Al_10_Ce_26_Pt_3_Ru_1_ ribbon had higher EOR activity and stability. A possible reason for this is that Cu can promote the formation of β-NiOOH with high catalytic activity and the unsaturated Ni and Ce ions provide sufficient oxygen vacancies, improving catalytic efficiency and anti-poisoning ability.

## Figures and Tables

**Table 1:** The initial potential of all electrocatalysts.

Dealloyed MG Ribbons After Activated (AD-MG Ribbons)	Initial Potentials (V)
AD-Cu_60_Al_10_Ce_26_Pt_3_Ru_1_	1.511
AD-Cu_40_Ni_20_Al_10_Ce_26_Pt_3_Ru_1_-1 h	1.475
AD-Cu_40_Ni_20_Al_10_Ce_26_Pt_3_Ru_1_-2 h	1.469
AD-Cu_40_Ni_20_Al_10_Ce_26_Pt_3_Ru_1_-3 h	1.437
AD-Cu_40_Ni_20_Al_10_Ce_26_Pt_3_Ru_1_-4 h	1.473

**Table 2:** ICP-MS results of the 0.05 M HNO_3_ acid aqueous solution after dealloying.

Ion conc. */ppb	Ni	Ce	Cu	Al	Pt	Ru
Cu_40_Ni_20_Al_10_Ce_26_Pt_3_Ru_1_ 0.05 M HNO_3,_ 3 h	42,101.2	123,896	84,455.7	10,750	79.278	263.962

* Ion conc.—ionic concentration. For dealloying, 0.02 g of metal ribbons were etched in 35 mL acid solutions.

## Data Availability

The original contributions presented in this study are included in the article and Supplementary Material. Further inquiries can be directed to the corresponding author.

## Supplementary Materials

The following supporting information can be downloaded at: https://www.mdpi.com/article/10.3390/ma18030701/s1, Figure S1: SEM-EDS spectra and element content of the As-spun Cu_60_Al_10_Ce_26_Pt_3_Ru_1_and Cu_40_Ni_20_Al_10_Ce_26_Pt_3_Ru_1_ MG ribbon; Figure S2: Selection EDS spectra of D-Cu_40_Ni_20_Al_10_Ce_26_Pt_3_Ru_1_-3 h ribbon; Figure S3: Selection EDS spectra of AD-Cu_40_Ni_20_Al_10_Ce_26_Pt_3_Ru_1_-3 h ribbon; Figure S4: Morphology of AD-Cu_40_Ni_20_Al_10_Ce_26_Pt_3_Ru_1_-3 h ribbon after EOR: (a–c) Surface SEM after 10 h CA test; (d) Selection EDS spectrum.; Table S1: The peak area percentages of O 1s calculated from the XPS spectrum.
