# Supplementary material for: Boosting Electrooxidation of Ethanol by Nickel Addition to Metallic Glass Ribbon Precursors
Source: Materials (Basel). 2025 Feb 5;18(3):701. doi: 10.3390/ma18030701 (PMC11821255; doi:10.3390/ma18030701)
Supplement: Supplementary file 1 [file materials-18-00701-s001.zip › materials-3376012-supplementary.pdf]

Supporting Information

Boosting Electrooxidation of Ethanol by Nickel Addition to Metallic Glass Ribbon Precursors

Jingjing Song [1,*], Bo Zhang [2], Yu Chen [3], Qingzhuo Hu [4], Fabao Zhang [4] and Langxiang Zhong [4]

[1] School of Materials and Chemical Engineering, Bengbu University, Bengbu 233030, China
[2] Songshan Lake Materials Laboratory, Dongguan 523808, China
[3] Anhui Haoou Electronic Technology Co., Ltd., Bengbu233010, China
[4] School of Materials Science and Engineering, Hefei University of Technology, Hefei 230009, China
* Correspondence: sjj@bbc.edu.cn; Tel.: +86-152-5520-2275

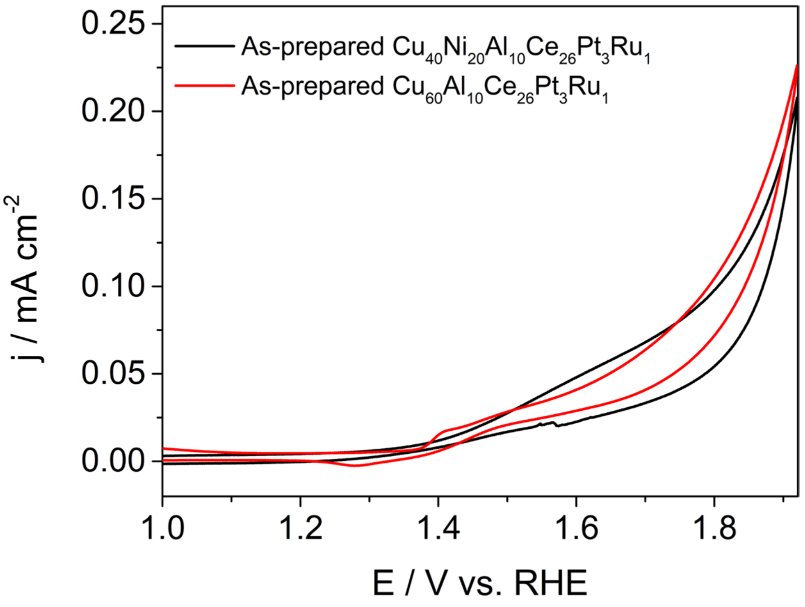

Figure S1. CV results of the as-spun $Cu_{40}Ni_{20}Al_{10}Ce_{26}Pt_3Ru_1$ and $Cu_{60}Al_{10}Ce_{26}Pt_3Ru_1$ MG ribbons performed in 1.0 M KOH + 1.0 M ethanol aqueous solution at a scan rate of 30 mV s^{-1}.

a

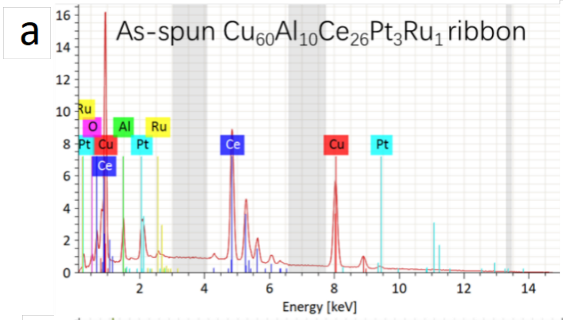

b

Element	At.No.	Mass Norm. [%]	Atom [%]	abs.error [%] (1 sigma)
Cu	29	46.51	59.83	1.45
Al	13	3.14	9.50	0.17
Ce	58	41.84	24.41	1.19
Pt	78	7.12	2.99	0.30
O	8	0.50	2.56	0.13
Ru	44	0.89	0.72	0.07
		100.00	100.00	

c

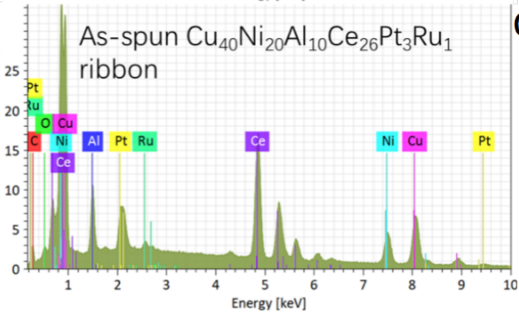

d

Element	At.No.	Mass Norm. [%]	Atom [%]	abs.error [%] (1 sigma)
C	6	0.00	0.00	0.00
O	8	0.73	3.53	0.17
Al	13	4.19	12.05	0.21
Ni	28	14.11	18.64	0.44
Cu	29	32.76	39.98	1.01
Ru	44	0.89	0.68	0.07
Ce	58	40.48	22.40	1.11
Pt	78	6.84	2.72	0.28
		100.00	100.00	

Figure S2 SEM-EDS spectra (a, c) and element content (b, d) of the As-spun $Cu_{60}Al_{10}Ce_{26}Pt_3Ru_1$ and $Cu_{40}Ni_{20}Al_{10}Ce_{26}Pt_3Ru_1$ MG ribbons.

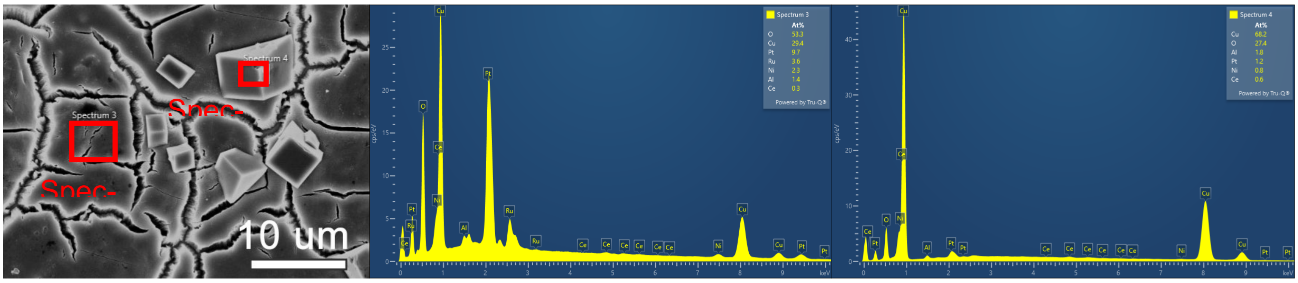

Figure S3. Selection EDS spectra of D-$Cu_{40}Ni_{20}Al_{10}Ce_{26}Pt_3Ru_1$-3 h ribbon.

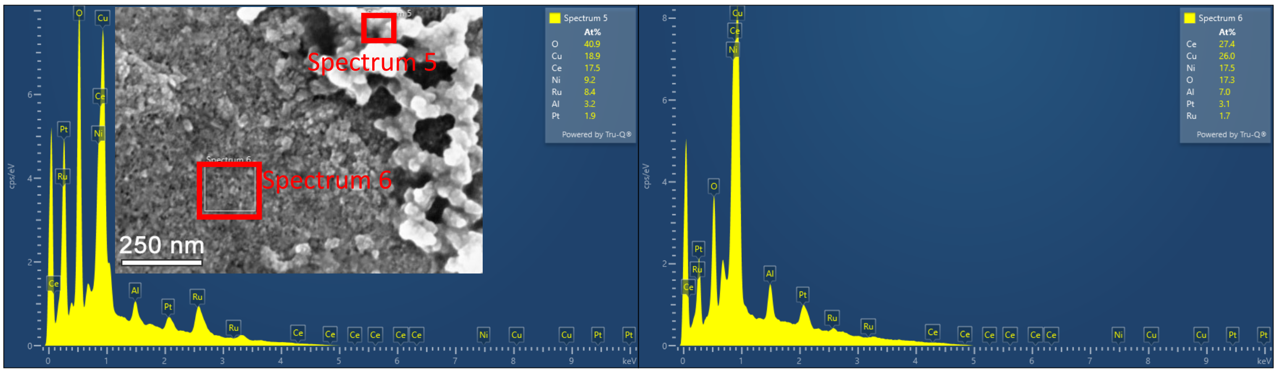

Figure S4. Selection EDS spectra of AD-$Cu_{40}Ni_{20}Al_{10}Ce_{26}Pt_3Ru_1$-3 h ribbon.

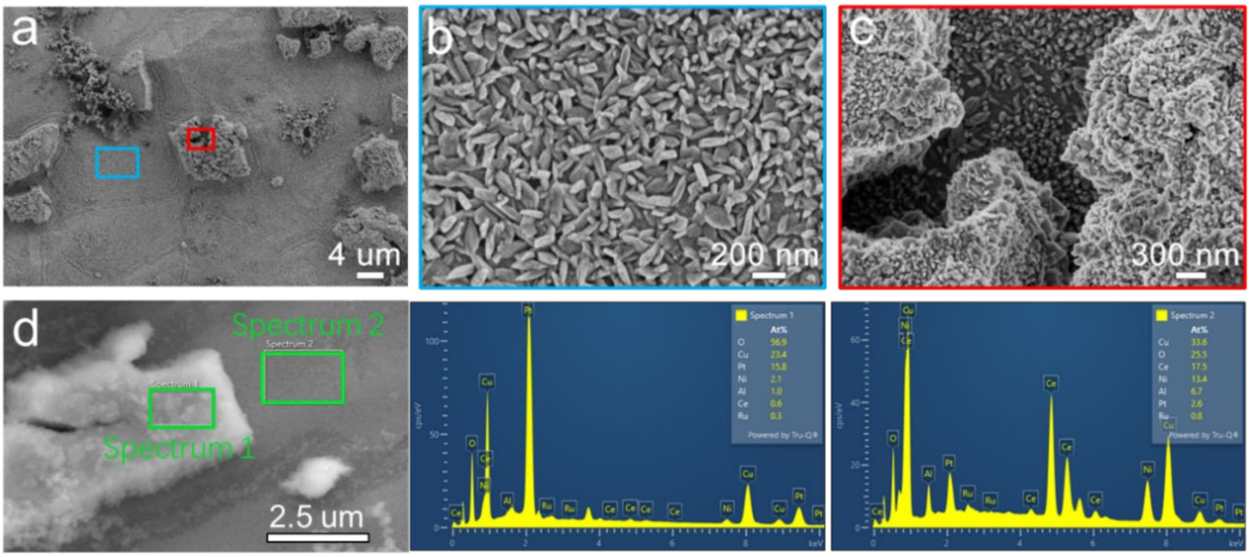

Figure S5. Morphology of AD-$Cu_{40}Ni_{20}Al_{10}Ce_{26}Pt_3Ru_1$-3 h ribbon after EOR: (a-c) Surface SEM after 10 h CA test; (d) Selection EDS spectrum.

Table S1. The peak area percentages of O 1s calculated from the XPS spectrums.

O	529.5	530.9	531.6	532.2
AD-$Cu_{40}Ni_{20}Al_{10}Ce_{26}Pt_3Ru_1$-3 h before EOR	20.82%	23.30%	25.89%	29.99%
AD-$Cu_{40}Ni_{20}Al_{10}Ce_{26}Pt_3Ru_1$-3 h after EOR	36.58%	21.96%	27.24%	14.22%
